# lncRNA SNHG3 promotes breast cancer progression by acting as a miR-326 sponge

**DOI:** 10.3892/or.2022.8255

**Published:** 2022-01-04

**Authors:** Haipeng Zhang, Na Wei, Wei Zhang, Lishennan Shen, Rongbo Ding, Qian Li, Simin Li, Ye Du

Oncol Rep 44: 1502-1510, 2020; DOI: 10.3892/or.2020.7690

Following the publication of the above article, the authors have requested that it be retracted. Subsequently to having performed these experiments, the authors have realized that their MCF-7 cells had become contaminated while performing the colony formation and transwell experiments. Furthermore, they were unable to reproduce the results from the *in vivo* experiments, and knockdown of *SNHG3* was found not to affect tumor growth in nude mice.

Following a further investigation in the Editorial Office, it also came to light that there were other possible anomalies associated with the presentation of the tumor images in Fig. 6B and the colony formation assay data in Fig. 2C. Therefore, theos article has been retracted from the Journal; all the authors agree to this retraction. The Editor and the authors would like to apologize for any inconvenience caused.

